# Epigenetic changes and their potential reversibility in mental health disorders

**DOI:** 10.1042/EBC20253020

**Published:** 2025-12-08

**Authors:** Micah Allen, Carlos Guerrero-Bosagna

**Affiliations:** 1 School of Medical Sciences, Faculty of Health, University of Victoria, Victoria, BC, Canada; 2 Physiology and Environmental Toxicology Program, Department of Organismal Biology, Uppsala University, Uppsala, Sweden

**Keywords:** epigenetics, mental illness, personalized medicine

## Abstract

Mental health disorder (MHD) incidence rates continue to rise, contributing significantly to the global disease burden. While their aetiology was once thought to be strictly genetic or environmental, the study of epigenetics has reshaped our understanding of their underlying mechanisms. Environmental exposures are understood as key players in the development of MHDs. Growing research has elucidated the critical role of environmental chemical exposures—particularly through endocrine-disrupting chemicals and heavy metals—in influencing MHD incidence through epigenetic mechanisms (i.e. DNA methylation, histone modification and non-coding RNA action). A key breakthrough in this field is the recognition that epigenetic modifications are not necessarily permanent. By exploiting the potential reversibility of DNA methylation and histone modification, new avenues for therapeutic interventions open, in which normal gene function could be restored. Understanding and harnessing epigenetic reversibility not only provides hope for novel and personalized treatment strategies but also underscores the importance of environmental protection policies in mental health prevention.

## Introduction

Mental health disorders (MHDs) are a leading cause of disability worldwide and contribute significantly to the global disease burden [1], ranking among the top 10 diseases globally. Individuals with severe mental illnesses have notably shorter life expectancies, often dying 10–20 years earlier than the general population [2,3]. Comorbidities among MHDs are common [4,5], with major depressive disorder (MDD) and generalized anxiety disorder (GAD) among the most frequent [6]. Such co-occurrences pose a diagnostic challenge, as symptoms like insomnia, difficulty concentrating, fatigue and agitation often overlap among multiple disorders, thus complicating the development of effective treatment plans [7]. Additionally, those who struggle with comorbid mental health conditions tend to have higher severity of illness, lower responses to treatments and interventions, poorer prognosis, lower quality of life and generally place more strain on the healthcare system [5,6]. However, these comorbidities extend beyond just GAD, with MDD frequently co-occurring with a range of other psychiatric conditions, such as post-traumatic stress disorder (PTSD), obsessive-compulsive disorder (OCD), eating disorders and substance use disorders [6,8]. Therefore, comorbidities present a significant challenge to both diagnosis and treatment, as symptoms intersect and interplay, which overall complicates the process. This highlights the importance of determining the underlying aetiology in order to identify the most effective treatment strategies.

While there has been a significant expansion of knowledge on MHDs, the causal mechanisms of psychiatric disorders remain largely elusive [9]. Early research on MHDs often focussed on identifying single genetic causes; however, advances in genetics have revealed a more nuanced understanding of heritability across disorders [9]. Genetic influence on MHDs is now largely described as a gradient, with more severe and less common disorders (e.g. autism, schizophrenia, bipolar disorder) contributing higher estimates of heritability, while more common and less severe disorders (e.g. anxiety, MDD) are associated with lower degrees of heritability [9,10]. Some argue that severe mental illnesses are a result of several genetic loci, while others dispute that they are rather associated with single genes [9]. However, most genetic variants that contribute to MHDs act in unison and are common occurrences [11]. Thus, every individual has a general genetic risk to a psychiatric disorder [11]. While genetic analysis has made significant strides in demonstrating its influence on MHDs, there are certain aspects of their development that are incongruent with a genetic explanation alone. For instance, multiple epidemiological studies have highlighted a strong association between low socioeconomic status and the occurrence of MHDs in children [12]. These relationships, which fall outside the scope of genetic explanations, underscore the importance of understanding environmental factors playing a causal role in the onset of mental health conditions.

### Epigenetics and environmental exposures

These previously unexplainable relationships can now be better understood through the lens of epigenetics. Once dominated by the nature vs. nurture debate, discourse surrounding MHDs has evolved to recognize that genetics and environment are not mutually exclusive. Rather, both interact in shaping the development and trajectory of phenotypes in general, including MHDs. This emphasizes the pivotal role of epigenetics in this dynamic and nuanced interplay [11]. Through processes such as DNA methylation, histone modification and non-coding RNA action, the chromatin state is altered, thereby influencing gene activation. Moreover, epigenetic modifications can respond to environmental exposures [13]. DNA methylation typically silences genes by adding methyl groups to cytosine bases neighbouring guanines; chemical modifications in histone tails can either loosen or tighten DNA winding around histones, affecting accessibility to transcription factors; and non-coding RNAs can interfere with transcription or translation processes [14]. Epigenetic patterns are established during crucial developmental periods in a cell type specific manner, molding tissue specific gene expression diversity.

Recognition of the profound impact of early life conditions on shaping health and disease risk has become widely acknowledged and increasingly emphasized, aligning with the principles of the Developmental Origins of Health and Disease (DOHaD) theory [12]. This well-established framework explains how the prenatal period, along with early-life environments and events, can result in the alteration of developmental trajectories [15]. These modifications may have lasting effects, influencing chronic health outcomes and disease susceptibility throughout the lifespan [15]. Thus, the origins and progression of MHDs can be further explained through the lens of DOHaD, with the ‘exposome’ representing the totality of early exposures and experiences during life [15]. Environmental exposures within the DOHaD theory include, but are not limited to, chemical and pharmacological, chronic stress and trauma, obesity and malnutrition, and other determinants of health during early life [15]. These environmental exposures have been studied extensively to understand their role in shaping MHDs.

### Early life exposures and MHDs

Growing amounts of empirical data suggest adverse childhood experiences (ACEs) increase the risk of MHDs, specifically MDD, PTSD, borderline personality disorder (BPD), GAD, suicidal ideation and dissociative symptoms [16–20]. Importantly, the risk of developing an MHD (specifically MDD), as well as their severity, is suggested to be dependent on the type of ACE. Furthermore, ACEs can act in single or clustered events, with the latter increasing severity [16,21]. ACEs predominantly associated with an increased risk of MHDs include sexual and physical abuse, childhood neglect, household dysfunction (such as parental psychiatric morbidity), as well as extreme traumatic shock (such as parental death) [16,17,21–24]. Thus, numerous ACEs have been shown to play a causal role in the incidence of MHDs throughout an individual’s lifetime. Importantly, these adverse experiences often coincide with different chemical exposures, which can exacerbate their impact on mental health outcomes. For instance, children living in impoverished communities often face disproportionate rates of chemical exposures, such as to lead or endocrine-disrupting chemicals (EDCs), which can hinder development and amplify the risks of MHDs [25].

Recent research highlights the potential link between early-child nutritional habits and MHDs, specifically obesity. Obesity has consequential impacts in children and adolescents on both physical health, mental health and quality of life. As the prevalence of childhood obesity has risen dramatically, so too has the need to better understand its impact not only on physical health, but also on the development of MHDs [26]. Research has found that overweight children experience difficulties with body image, bullying, peer-peer relationships, lower self-esteem and self-perception, lower school performance and lower health-related quality of life (HRQoL), all of which are risk factors for developing MHDs in adolescence or in adulthood [26–31]. Additionally, psychiatric comorbidities within childhood obesity are vast, including a range of mood and anxiety disorders, substance use, as well as somatoform and eating disorders [32]. Thus, connections can be drawn between childhood obesity and the increased risk of developing MHDs. In parallel, researchers have theorized how certain environmental chemical exposures can induce obesity. These are known as obesogens, which act at critical developmental periods, causing metabolic disturbances and increasing obesity susceptibility [33–35]. There are several different major classes and sources of obesogens: bisphenols, phthalates, parabens, non-steroid oestrogens, brominated flame retardants, polychlorinated biphenyls, organotins, all of which, upon exposure at work, home or through the environment, increase the risk of obesity. Importantly, exposure to obesogens may in turn heighten the risk of developing a MHD [34].

The DOHaD framework now extends to environmental exposures to maternal prescribed medications, psychotropic substances, stimulant drugs or alcohol prenatally or during early life. Individuals exposed to these compounds have heightened risk for both developmental disorders and MHDs [36–39], specifically those related to mood, anxiety and behavioural disorders [40]. Of those listed, foetal alcohol spectrum disorder remains the most studied, with heavy prenatal alcohol exposure meeting the criteria for at least one psychiatric disorder, namely MDD, BPD, GAD and substance use disorders [39,41,42]. As both addictive and essential drug use during pregnancy rises, so too has the awareness of its detrimental effects on fetuses, such as neurodevelopmental delays, poor physical health and, notably, an increased risk of MHDs [38,43]. Importantly, children prenatally affected by addictive drugs are also more likely to experience additional risk factors when a parent has a substance use disorder. These risk factors include poverty, parental co-morbidities, household dysfunction and poor overall health, all of which can indirectly contribute to the incidence of MHDs [38,43].

In addition to the effects observed during lifetime, recent research has uncovered effects of environmental exposures on behaviour across generations. One study that exemplifies this phenomenon examined the inheritance of parental trauma in offspring [44]. They exposed F0 mice to a fear-conditioning odour before conception and discovered that conceived F1 and F2 generations had increased behavioural sensitivity to the F0-conditioned odour, but not to other odours [44]. DNA methylation changes were found in olfactory receptor genes in the sperm of the F0 and F1 generations, highlighting that the transmission of the behavioural effects across generations may be mediated by germ line epigenetic alterations [44]. Another study examined microRNA (miRNA) expression in mice and its progeny upon exposure to traumatic stress in early life [45]. Researchers found that traumatic experiences in early life led to the alterations of miRNA in the serum, brain and sperm of traumatized mice and their progenies. Injecting sperm RNAs from traumatized males into wildtype oocytes reproduced the behavioural and metabolic alterations in resulting offspring [45]. Together, these studies exemplify an important area in the study of epigenetics, displaying how epigenetic modifications can help explain the molecular mechanisms involved in the transgenerational transmission of traumatic events.

### Different sources of environmental chemical exposures

The consequences of environmental chemical exposures on the etiology of a variety of diseases and conditions—including cancer, premature birth, infertility and neurodevelopmental disorders—are well documented, particularly due to their roles in endocrine disruption and neurotoxicity [46–48]. However, their influence on MHD incident rates remains less understood.

### Endocrine-disrupting chemicals

EDCs are a large class of exogenous chemicals that interfere with any hormone action through various mechanisms: disruption of hormone synthesis, transport or breakdown, alteration of the development of hormone receptors, modification of hormone binding, or action as hormone antagonists [46,48–50]. Many environmental EDCs are released to the environment during manufacturing, production and use of synthetic man-made materials like pesticides, plastics, flame-retardants, metals, food additives and personal care products [46,48,49]. Particularly, there are growing concerns around the influence that EDC exposures, especially in early life, have on MHD outcomes and incident rates, as EDC effects are now far reaching [51].

Although EDCs encompass approximately 85,000 chemical compounds of different types, only 1000 are recognized and understood as having endocrine-disrupting properties [52,53]. These include, but are not limited to, phenols (bisphenol A [BPA] and its analogues), phthalates, pre- and polyfluoroalkyl substances (PFASs), polycyclic aromatic hydrocarbons (PAHs), alkylphenols, brominated flame retardants, halogenated aromatics and pesticides [48]. The links between various EDCs and internalizing psychiatric symptoms (ex. anxiety, depression and somatisation) or externalizing psychiatric symptoms (ex. aggression, hyperactivity and conduct problems), as well as MDD, have become increasingly evident [48,52,54–57]. Certain EDCs have been shown to disrupt and dysregulate the hypothalamic-pituitary-adrenal and the hypothalamic–pituitary–gonadal axes, both of which are critical for neuronal processes and mental health [55,58]. Such disruption can impair stress regulation, hormone balance and brain development, contributing to mood disorders, cognitive impairments and other neuropsychological conditions [55,58].

Even at low doses, EDCs can influence bodily systems, sometimes with impacts only observed later in life [48]. Epigenetic markers such as DNA methylation can potentially aid in the explanation of latency in neurobehavioural disorders upon EDC exposure [53]. Additionally, DNA methylation changes resulting from EDC action have been implicated in the aetiology of neurological disorders and MHDs, especially when occurring on genes involved in serotonin and dopamine pathways [59,60]. For example, one study exposed adolescent male rats to chlorpyrifos , an organophosphorus pesticide, and then subjected the rats to a variety of emotional behavioural tests related to serotonergic function [61]. Researchers found that repeated exposure to chlorpyrifos led to alterations of emotional behaviours related to serotonergic system [61]. One way that EDCs can impact DNA methylation is by altering the expression of DNA methyltransferases (DNMT), enzymes that add methyl groups to cytosines neighbouring guanines (CpGs). It is hypothesized that EDC action on DNMT expression is a main driver of downstream epigenetic and cellular changes [14]. Kundakovic *et al*. showed that BPA exposure altered mRNA levels of DNMT1, DNMT3A and oestrogen receptors in the cortex and hypothalamus of mice, leading to social and anxiety-like behaviour [62,63]. Even at low dose exposure during early life, EDCs can cause incomplete and impaired methylation of specific gene regions, hindering hippocampal neurogenesis and overall brain function, thus bestowing greater susceptibility to neuronal disorders such as MDD [55,59]. Many EDCs are small, lipophilic compounds, rendering them the ability to diffuse through membranes and even cross the blood-brain barrier [55,64]. Thus, EDCs can act on various receptors, although its influence on the nuclear receptor family (e.g. oestrogen receptors, androgen receptors and thyroid hormone receptors) is the most studied mode of action [14]. Additionally, agonistic or antagonistic EDC action on the aryl hydrocarbon receptor has gained recognition, as its activation by persistent organic pollutants (POP), such as pesticides, regulates gene transcription and expression in a similar way as nuclear receptors [14,65–67]. POP exposure during early life can cause improper brain formation and behavioural or cognitive defects, mediated through aryl hydrocarbon receptor [59]; this is exemplified in individuals working in environments contaminated with pesticides (e.g. chlorpyrifos, paraquat) and polychlorinated biphenyls, who exhibit symptoms of depression and anxiety, as well as learning deficits and memory dysfunction [59,68]. In addition to DNA methylation, EDC exposure can alter other epigenetic mechanisms, such as histone-modifying enzyme activity [14,69,70]. For example, upon exposure to the pesticide TDCC, the activity of histone methyltransferases can be amplified, thus modifying the epigenetic status, with cellular consequences that impact pathology [65]. Recent data have also shown that EDC exposure can alter miRNA expression, cumulatively contributing to the array of epigenetic modifications involved in the development of depression [59,64,71].

EDCs impact on neurodevelopment and other neurological processes occur primarily through changes in its monoaminergic transmission [59,72,73]. EDCs (e.g. BPA) affect this system, as well as the mesolimbic dopamine pathway, via the disruption of dopamine synthesis, release and turnover, which impacts its transport and expression upon receptor binding [59,70,74–76]. Consequently, EDC exposure would increase depression incidents due to the alterations in neurotransmitter systems [59,61]. Importantly, environmental chemical exposures rarely occur in isolation but rather in an intertwined complex system where multiple different exposures happen simultaneously [40]. These cocktails of exposures are pervasive, ubiquitous and continuously impact our mental health, contributing to the onset of symptoms or diagnoses of MHDs.

### Heavy metals

The relationship between early-life exposure and the toxic effects of heavy metals on neurodevelopmental outcomes is well established and known to contribute to intellectual disabilities, autism spectrum disorder (ASD), learning difficulties and modest IQ reductions [40,77]. Heavy metals are widespread chemical contaminants that can be extremely toxic to several organ systems and are associated with pathologies such as cancer, cardiovascular, neurological and autoimmune diseases [40,78]. As epidemiological and clinical studies continue to grow, the links between heavy metals and MHDs become more established [40,77]. Research has signalled that heavy metal exposure, such as lead, cadmium, manganese, arsenic and zinc, is associated with various mental illnesses, including depression, anxiety, cognitive dysfunction, mood disorders, bipolar disorder and schizophrenia [79–81]. Blood lead concentrations in children have declined substantially worldwide, dropping from 15 µg/dl in 1970 to approximately 2.0 µg/dl by the late 1990s [82]. However, average levels of lead and other heavy metals vary across countries, largely influenced by differences in regulatory frameworks, waste management practices and infrastructure [82]. Notably, even blood lead concentrations below 5 µg/dl have been associated with reductions in IQ, as well as increased risks of behavioural disorders and emotional dysregulation [82–84]. One study applied a posterior predictive distribution to assess cord blood heavy metals in relation to ASD and identified threshold levels of exposure that can predict future ASD incidence [85]. Thus, heavy metals can act as biomarkers that predict neurodevelopmental disorders such as ASD, galvanizing researchers to investigate whether heavy metals like lead or cadmium can become additional biomarkers in the diagnosis of psychiatric disorders.

The toxic action of heavy metals on the brain is unique albeit concerning, as it can damage the blood–brain barrier, reduce neuronal plasticity, induce oxidative stress and neuroinflammation, and importantly, modify gene expression patterns leading to increased vulnerability to MHDs [40]. Metal ions like lead and cadmium can generate reactive oxygen species , which is hypothesized to be one of the main drivers of epigenetic alterations upon heavy metal exposure [78,80,86]. These reactive oxygen species accumulate and can affect epigenetic markers in different ways, for example, by interfering with the ability of methyltransferases to interact with DNA [78,86] or by silencing DNA repair and tumour-suppressor genes [87]. It has been reported that arsenic, nickel, chromium, cadmium and methylmercury modify the DNA methylation status of a variety of genes [78,87–90]. Additionally, arsenic, nickel, chromium, methylmercury and copper have been shown to elicit changes in histone modifications, while arsenic, aluminium and cadmium are reported to impact non-coding RNA expression [78,87–89]. In parallel, epigenetic research on depression has demonstrated abnormal DNA methylation patterns related to neurotransmission, particularly in serotonin and dopamine pathways [60], while highlighting the roles of histone modifications and non-coding RNA action [78,86] in synaptic adaptability and stress response [60]. Together, these mechanisms play a crucial role in shaping MHD outcomes; understanding their patterns and functions could position epigenetic modifications as potential therapeutic targets.

### The potential of epigenetic reversibility

Epigenetic changes can be small, potentially cumulative and may develop over time. One example of this is epigenetic clocks, which is a prediction model that approximates the chronological age of different tissues and cell types with high accuracy [91]. Epigenetic clocks measure the cumulative effects of an epigenetic maintenance system by quantifying DNA methylation at specific CpG sites [91]. Environmental exposures such as organochloride pesticide and benzene, trichlorethylene and tobacco smoke exposure have been associated with greater epigenetic age acceleration, as these exposures have altered DNA methylation patterns that develop over time [92,93]. Different levels of epigenetic regulation can interact in a dynamic, intricate and interconnected way that has the potential to generate a self-reinforcing cycle of epigenetic events.

Currently, there are ongoing discussions about how permanent epigenetic changes are, as well as their potential to be reverted when associated with diseases. Speculation surrounding the potential reversibility of aberrant epigenetic modifications is only rudimentarily understood, warranting new research to explore this. Over two decades ago, the rousing publication from biologists Ringrose and Paro, ‘Remembering Silence’, prompted conversations that questioned the ability of epigenetic modifications to return to their silenced state [94]. If one was exposed to a traumatic event and thus acquired a pathological epigenetic modification, could they ultimately rid themselves of this modification? Although ‘Remembering Silence’ pertained to epigenetic traits following a traumatic event, the same question resides following environmental chemical exposures.

**Figure 1:**
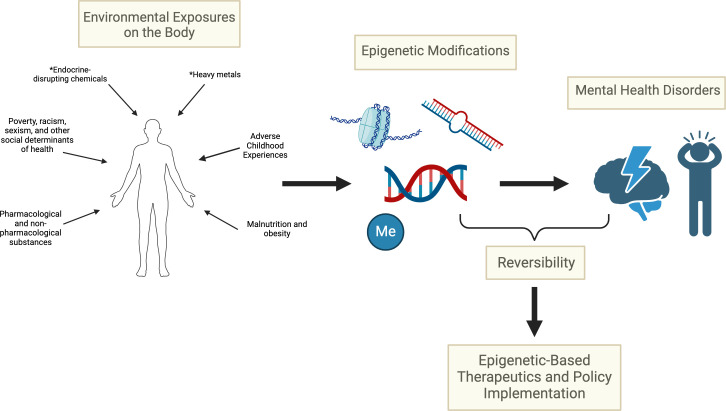
Schematic overview depicting how environmental exposures can cause epigenetic alterations through the action of DNA methylation, non-coding RNA action and histone modification, leading to an increase in mental health disorder incidence. The potential for these epigenetic modifications to be largely reversed can be exploited to alleviate mental health disorder burden and symptoms. This reversibility can provide new avenues for therapeutics and tools for policymakers. The * represents an environmental chemical exposure. Created in (95,113 ) .

The reversibility of epigenetic modifications presents exciting and novel avenues for personalized medicine in MHD treatments, potentially offering respite to individuals with MHD influenced by environmental chemical exposures—either by targeting the specific epigenetic modifications, or instead tailoring treatment strategies based on the distinct epigenetic profiles (Figure 1). This is particularly relevant when considering that the brain’s susceptibility to epigenetic modifications—such as the dynamic processes of DNA methylation [95] and histone modifications [96–98]—plays a key role in neuronal plasticity and its stress response pathways to the environment [60,95]. Unique variations in epigenetic patterns can provide opportunities for personalized treatment that targets genes associated with plasticity and synaptic activity [60]. If tissue-specific epigenetic modifications (e.g. DNA methylation) can be reversed to pre-exposure levels, either through pharmacological or non-pharmacological interventions, or removal of pathology-causing exposures, significant breakthroughs in treatment options for individuals with MHDs can occur.

Despite the potential of epigenetic reversibility for personalized medicine in psychiatric disorders, not much is known about its mechanism. Studies in lab animals have shown that reversibility is possible. For example, a study showed that DNA methylation changes associated with smoking were largely reversed after cessation [99]. Another study assessing epigenetic modifications in response to heavy metal exposures found that removing nickel treatment led to the re-expression of a nickel-silenced gene [87]. Overall, these studies highlight the potential for similar reversibility in other exposure-related epigenetic changes. However, although reversibility is possible, the developmental time in which exposures take place plays an important role in the ability of epigenetic changes to be preserved or reversed. For instance, in mice hippocampus, one study found that DNA methylation changes were reversible in early life but became fixed and irreversible in adult life [100], suggesting that early-life environment is impactful on adult brain methylation pattern [101].

Epigenetic changes have also been shown to respond not only to chemical exposures but also to behavioural interventions. Kripalani et al. [102] examined DNA methylation changes after mind–body therapies such as meditation, yoga and tai-chi, and found that DNA methylation was lower in those who participated in mind–body therapies compared with those who did not, explaining the potential for DNA methylation patterns to decrease, and thus reverse in later life if one engages in positive behavioural experiences. Similarly, a different study employed a one-week intensive resident group program for adolescents who experienced multiple ACE that included mindfulness training, artistic expression and EMDR group therapy. A decrease in PTSD-related symptoms and changes in DNA methylation levels was found to correlate with improvements in trauma-related psychological measures [103]. Besides changes in DNA methylation triggered by behavioural interventions, epigenetic manipulations can also change behaviour. For example, one study found that by inhibiting histone deacetylase, the effects of chronic social defeat stress in mice were reversed, reducing histone-H3 acetylation and exhibiting actions similar to the antidepressant fluoxetine [101,104]. Together, these findings suggest that targeted interventions can modulate epigenetic patterns, indicating the potential reversibility that these consequential epigenetic modifications have on behaviour, offering new approaches to therapeutics.

Some drugs utilized to treat psychiatric disorders are known to have an epigenetic effect [105], highlighting the concept of epigenetic reversibility in a therapeutical context. This opens the possibility to create novel drugs that target epigenetic modifications that are known to associate with behavioural phenotypes. The emerging field of epigenetic therapeutics suggests that designing treatments specifically aimed at reversing epigenetic changes associated with detrimental outcomes may be possible, resulting in more efficient treatment alternatives. Epidrugs—a growing class of drugs designed to selectively modify the epigenetic landscape—target aberrant epigenetic modifications by inhibiting or reprogramming dysregulated epigenetic enzymes [106,107]. Initially developed for cancer therapy [106], epidrugs are now being investigated as potential treatments for central nervous system disorders, including MHDs [107]. For instance, epidrugs can be utilized as potential antidepressants to exploit the reversibility of abnormal epigenetic modifications associated with depression [60,108]. Epidrugs, such as DMNT inhibitors or histone deacetylase inhibitors, have shown therapeutic potential but may also produce off-target effects, disrupting the homeostatic function of healthy cells and causing adverse effects [109,110]. To minimize these off-target toxicities while enhancing specificity and efficacy, researchers have been investigating novel delivery mechanisms and exploring epigenetic biomarkers to support the screening, diagnosis and development of these targeted treatments [110]. Currently, FDA-approved epidrugs are used in combination with histone deacetylase inhibitors, with most clinical success and approved applications seen in the treatment of haematological malignancies [111]. For example, the FDA-approved drug Decitabine is a methyltransferase inhibitor that, along with other functions, targets DNA methyltransferase 1 to treat myelodysplastic syndrome and acute myeloid leukaemia [112]. If correctly targeted, epidrugs could modulate epigenetic marks associated with chronic stress, neuroinflammation and synaptic plasticity, potentially reverting abnormal epigenetic patterns linked to MHDs and alleviating its associated symptoms [60].

Not only could epigenetic reversibility significantly influence patient treatment plans, but it could also positively impact policy. If certain chemical-induced epigenetic changes can be reversed upon exposure removal, this can inform policy makers, as well as the public's opinion, and can also garner additional legitimacy to policy makers when building their cases to address these problems [113]. Recognizing the epigenetic impact of pollutants, endocrine disruptors and heavy metals strengthens the case for stricter regulations on hazardous substances, thus reducing public health risks.

Understanding these reversible changes can empower and offer hope to individuals by enabling targeted interventions and developing selective pharmacological agents that modify these epigenetic changes. One aspect would be to employ these therapies in patients showing treatment resistance, as it has been suggested that epigenetic changes contribute to this [95,114]. Exploiting the reversibility of epigenetic modifications could not only improve patient outcomes for MHDs associated with environmental chemical exposures but also reinforces the importance of reducing these exposures through both medical and policy-driven approaches.

**Summary points**

- Environmental exposures in early life, such as adverse childhood experiences, child obesity and pharmacological and non-pharmacological substances, are impactful in the onset and aetiology of mental health disorders (MHDs) in later life.
- Knowledge about environmental chemical exposures, which includes endocrine-disrupting chemicals and heavy metals, continues to grow as it illustrates its negative effects exerted on the body. The epigenetic mechanisms of these environmental chemical exposures can, in part, aid in describing its contribution to increased MHD incidence rates.
- The ability for epigenetic mechanisms, particularly DNA methylation, to be reversed remained largely elusive. Several studies have described the potential for DNA methylation patterns to decrease or reverse altogether upon cessation of stimulus or positive experiences. Epigenetic reversibility provides new avenues for targeted therapeutics and environmental policy.

## Open Access

This article has been published open access under our Subscribe to Open programme, made possible through the support of our subscribing institutions, learn more here: https://portlandpress.com/pages/open_access_options_and_prices#conditional

## Abbreviations

ACEs: Adverse Childhood Experiences
ASD: Autism Spectrum Disorders
BPA: Bisphenol A
BPD: Borderline Personality Disorder
DNMT: DNA methyltransferases
DOHaD: Developmental Origins of Health and Disease
EDCs: Endocrine Disrupting Chemicals
GAD: generalized anxiety disorder
MDD: major depressive disorder
MHD: mental health disorder
OCD: obsessive-compulsive disorder
POP: Persistent Organic Pollutants
PTSD: post-traumatic stress disorder

## References

[1] Wu Y. Wang L. Tao M. Cao H. Yuan H. Ye M. et al. 2023 Changing trends in the global burden of mental disorders from 1990 to 2019 and predicted levels in 25 years Epidemiol. Psychiatr. Sci. 32 e63 10.1017/S2045796023000756 37933540 PMC10689059

[2] Mental health 2025 Mental health https://www.who.int/health-topics/mental-health

[3] Husky M.M. Alvarez Fernandez V. Tapia G. Oprescu F. Navarro-Mateu F. Kovess-Masfety V 2020 Mental disorders and medical conditions associated with causing injury or death: A population-based study Psychiatry Res 287 112899 10.1016/j.psychres.2020.112899 32169724

[4] Wu L.T. Kouzis A.C. Leaf P.J 1999 Influence of comorbid alcohol and psychiatric disorders on utilization of mental health services in the national comorbidity survey AJP 156 1230 1236 10.1176/ajp.156.8.1230 10450265

[5] McGrath J.J. Lim C.C.W. Plana-Ripoll O. Holtz Y. Agerbo E. Momen N.C. et al. 2020 Comorbidity within mental disorders: a comprehensive analysis based on 145 990 survey respondents from 27 countries Epidemiol. Psychiatr. Sci. 29 e153 10.1017/S2045796020000633 32782057 PMC7443806

[6] Al-Asadi A.M. Klein B. Meyer D 2015 Multiple comorbidities of 21 psychological disorders and relationships with psychosocial variables: a study of the online assessment and diagnostic system within a web-based population J. Med. Internet Res. 17 e55 10.2196/jmir.4143 25803420 PMC4392551

[7] Zbozinek T.D. Rose R.D. Wolitzky-Taylor K.B. Sherbourne C. Sullivan G. Stein M.B. et al. 2012 Diagnostic overlap of generalized anxiety disorder and major depressive disorder in a primary care sample Depress. Anxiety 29 1065 1071 10.1002/da.22026 23184657 PMC3629816

[8] Fava M. Alpert J.E. Carmin C.N. Wisniewski S.R. Trivedi M.H. Biggs M.M. et al. 2004 Clinical correlates and symptom patterns of anxious depression among patients with major depressive disorder in STAR*D Psychol. Med. 34 1299 1308 10.1017/s0033291704002612 15697056

[9] Uher R. Zwicker A 2017 Etiology in psychiatry: embracing the reality of poly-gene-environmental causation of mental illness World Psychiatry 16 121 129 10.1002/wps.20436 28498595 PMC5428165

[10] Polderman T.J.C. Benyamin B. De Leeuw C.A. Sullivan P.F. Van Bochoven A. Visscher P.M et al. 2015 Meta-analysis of the heritability of human traits based on fifty years of twin studies Nat. Genet. 47 702 709 10.1038/ng.3285 25985137

[11] Andreassen O.A. Hindley G.F.L. Frei O. Smeland OB 2023 New insights from the last decade of research in psychiatric genetics: discoveries, challenges and clinical implications World Psychiatry 22 4 24 10.1002/wps.21034 36640404 PMC9840515

[12] Reiss F. Meyrose A.-K. Otto C. Lampert T. Klasen F. Ravens-Sieberer U 2019 Socioeconomic status, stressful life situations and mental health problems in children and adolescents: Results of the German BELLA cohort-study PLoS ONE 14 e0213700 10.1371/journal.pone.0213700 30865713 PMC6415852

[13] Grezenko H. Ekhator C. Nwabugwu N.U. Ganga H. Affaf M. Abdelaziz A.M. et al. 2023 Epigenetics in neurological and psychiatric disorders: a comprehensive review of current understanding and future perspectives Cureus 15 e43960 10.7759/cureus.43960 37622055 PMC10446850

[14] Alavian-Ghavanini A. Rüegg J 2018 Understanding epigenetic effects of endocrine disrupting chemicals: from mechanisms to novel test methods Basic Clin. Pharmacol. Toxicol. 122 38 45 10.1111/bcpt.12878 28842957

[15] Hagemann E. Silva D.T. Davis J.A. Gibson L.Y. Prescott S.L 2021 Developmental origins of health and disease (DOHaD): the importance of life-course and transgenerational approaches Paediatr. Respir. Rev 40 3 9 10.1016/j.prrv.2021.05.005 34148804

[16] Chapman D.P. Whitfield C.L. Felitti V.J. Dube S.R. Edwards V.J. Anda R.F 2004 Adverse childhood experiences and the risk of depressive disorders in adulthood J. Affect. Disord. 82 217 225 10.1016/j.jad.2003.12.013 15488250

[17] Dunn E.C. Nishimi K. Powers A. Bradley B 2017 Is developmental timing of trauma exposure associated with depressive and post-traumatic stress disorder symptoms in adulthood? J. Psychiatr. Res 84 119 127 10.1016/j.jpsychires.2016.09.004 27728852 PMC5479490

[18] Jiang C. Jiang S 2022 Effects of adverse childhood experiences on late-life mental health: potential mechanisms based on a nationally representative survey in china Arch. Gerontol. Geriatr. 100 104648 10.1016/j.archger.2022.104648 35134613

[19] Dube S.R. Anda R.F. Felitti V.J. Chapman D.P. Williamson D.F. Giles W.H 2001 Childhood abuse, household dysfunction, and the risk of attempted suicide throughout the life span: findings from the adverse childhood experiences study JAMA 286 3089 3096 10.1001/jama.286.24.3089 11754674

[20] Sidamon-Eristoff A.E. Cohodes E.M. Gee D.G. Peña C.J 2022 Trauma exposure and mental health outcomes among Central American and Mexican children held in immigration detention at the United States-Mexico border Dev. Psychobiol. 64 e22227 10.1002/dev.22227 35050507

[21] Björkenstam E. Vinnerljung B. Hjern A 2017 Impact of childhood adversities on depression in early adulthood: a longitudinal cohort study of 478,141 individuals in sweden J. Affect. Disord 223 95 100 10.1016/j.jad.2017.07.030 28735168

[22] Wirback T. Möller J. Larsson J.-O. Galanti M.R. Engström K 2014 Social factors in childhood and risk of depressive symptoms among adolescents--a longitudinal study in Stockholm, Sweden Int. J. Equity Health 13 96 10.1186/s12939-014-0096-0 25384415 PMC4243322

[23] Rajalin M. Hirvikoski T. Renberg E.S. Åsberg M. Jokinen J 2020 Exposure to early life adversity and interpersonal functioning in attempted suicide Front. Psychiatry 11 552514 10.3389/fpsyt.2020.552514 33093835 PMC7527599

[24] Ceccarelli C. Prina E. Muneghina O. Jordans M. Barker E. Miller K. et al. 2022 Adverse childhood experiences and global mental health: avenues to reduce the burden of child and adolescent mental disorders Epidemiol. Psychiatr. Sci. 31 e75 10.1017/S2045796022000580 36245402 PMC9583628

[25] Gladieux M. Gimness N. Rodriguez B. Liu J 2023 Adverse childhood experiences (ACEs) and environmental exposures on neurocognitive outcomes in children: empirical evidence, potential mechanisms, and implications Toxics 11 259 10.3390/toxics11030259 36977024 PMC10055754

[26] Ottova V. Erhart M. Rajmil L. Dettenborn-Betz L. Ravens-Sieberer U 2012 Overweight and its impact on the health-related quality of life in children and adolescents: results from the European KIDSCREEN survey Qual. Life Res. 21 59 69 10.1007/s11136-011-9922-7 21557001

[27] Russell-Mayhew S. McVey G. Bardick A. Ireland A 2012 Mental health, wellness, and childhood overweight/obesity J. Obes. 2012 281801 10.1155/2012/281801 22778915 PMC3388583

[28] Förster L.-J. Vogel M. Stein R. Hilbert A. Breinker J.L. Böttcher M. et al. 2023 Mental health in children and adolescents with overweight or obesity BMC Public Health 23 135 10.1186/s12889-023-15032-z 36658514 PMC9849834

[29] Griffiths L.J. Parsons T.J. Hill A.J 2010 Self-esteem and quality of life in obese children and adolescents: a systematic review Int. J. Pediatr. Obes. 5 282 304 10.3109/17477160903473697 20210677

[30] Braet C. Mervielde I. Vandereycken W 1997 Psychological aspects of childhood obesity: a controlled study in a clinical and nonclinical sample J. Pediatr. Psychol. 22 59 71 10.1093/jpepsy/22.1.59 9019048

[31] De Beer M. Hofsteenge G.H. Koot H.M. Hirasing R.A. Delemarre-van de Waal H.A. Gemke R.J.B.J 2007 Health-related-quality-of-life in obese adolescents is decreased and inversely related to BMI Acta Paediatr. 96 710 714 10.1111/j.1651-2227.2007.00243.x 17381471

[32] Vila G. Zipper E. Dabbas M. Bertrand C. Robert J.J. Ricour C. et al. 2004 Mental disorders in obese children and adolescents Psychosom. Med. 66 387 394 10.1097/01.psy.0000126201.12813.eb 15184702

[33] Heindel J.J. Lustig R.H. Howard S. Corkey B.E 2024 Obesogens: a unifying theory for the global rise in obesity Int. J. Obes. (Lond) 48 449 460 10.1038/s41366-024-01460-3 38212644 PMC10978495

[34] Heindel J.J. Howard S. Agay-Shay K. Arrebola J.P. Audouze K. Babin P.J et al. 2022 Obesity II: establishing causal links between chemical exposures and obesity Biochem. Pharmacol 199 115015 10.1016/j.bcp.2022.115015 35395240 PMC9124454

[35] Mohajer N. Du C.Y. Checkcinco C. Blumberg B 2021 Obesogens: how they are identified and molecular mechanisms underlying their action Front. Endocrinol. (Lausanne) 12 780888 10.3389/fendo.2021.780888 34899613 PMC8655100

[36] Carlezon W.A. Konradi C 2004 Understanding the neurobiological consequences of early exposure to psychotropic drugs: linking behavior with molecules Neuropharmacology 47 Suppl 1 47 60 10.1016/j.neuropharm.2004.06.021 15464125 PMC4204484

[37] Dreier J.W. Bjørk M.-H. Alvestad S. Gissler M. Igland J. Leinonen M.K. et al. 2023 Prenatal exposure to antiseizure medication and incidence of childhood- and adolescence-onset psychiatric disorders JAMA Neurol. 80 568 577 10.1001/jamaneurol.2023.0674 37067807 PMC10111234

[38] Oei J.L 2024 Improving neurological and mental health outcomes for children with prenatal drug exposure Semin. Fetal Neonatal Med 29 101557 10.1016/j.siny.2024.101557 39537449

[39] Crocker N. Fryer S. Mattson S 2013 Second. John Wiley & Sons, Inc Exposure to Teratogens as a Risk Factor for Psychopathology. Second. Child And Adolescent Psychopathology https://perpus.univpancasila.ac.id/repository/EBUPT180233.pdf#page=295

[40] James A.A. OShaughnessy KL 2023 Environmental chemical exposures and mental health outcomes in children: a narrative review of recent literature Front. Toxicol. 5 1290119 10.3389/ftox.2023.1290119 38098750 PMC10720725

[41] Pei J. Denys K. Hughes J. Rasmussen C 2011 Mental health issues in fetal alcohol spectrum disorder J. Ment. Health 20 438 448 10.3109/09638237.2011.577113 21780939

[42] Georgieff M.K. Tran P.V. Carlson E.S 2018 Atypical fetal development: Fetal alcohol syndrome, nutritional deprivation, teratogens, and risk for neurodevelopmental disorders and psychopathology Dev. Psychopathol. 30 1063 1086 10.1017/S0954579418000500 30068419 PMC6074054

[43] Eriksson M. Larsson G. Zetterström R 1979 Abuse of alcohol, drugs and tobacco during pregnancy--consequences for the child Paediatrician 8 228 242 492732

[44] Dias B.G. Ressler K.J 2014 Parental olfactory experience influences behavior and neural structure in subsequent generations Nat. Neurosci. 17 89 96 10.1038/nn.3594 24292232 PMC3923835

[45] Gapp K. Jawaid A. Sarkies P. Bohacek J. Pelczar P. Prados J. et al. 2014 Implication of sperm RNAs in transgenerational inheritance of the effects of early trauma in mice Nat. Neurosci. 17 667 669 10.1038/nn.3695 24728267 PMC4333222

[46] Kumar M. Sarma D.K. Shubham S. Kumawat M. Verma V. Prakash A. et al. 2020 Environmental endocrine-disrupting chemical exposure: role in non-communicable diseases Front. Public Health 8 553850 10.3389/fpubh.2020.553850 33072697 PMC7541969

[47] Predieri B. Iughetti L. Bernasconi S. Street M.E 2022 Endocrine disrupting chemicals’ effects in children: what we know and what we need to learn? Int. J. Mol. Sci. 23 11899 10.3390/ijms231911899 36233201 PMC9570268

[48] Özel F. Rüegg J 2023 Exposure to endocrine-disrupting chemicals and implications for neurodevelopment Dev. Med. Child Neurol. 65 1005 1011 10.1111/dmcn.15551 36808586

[49] Jacobs M.N. Marczylo E.L. Guerrero-Bosagna C. Rüegg J 2017 Marked for life: epigenetic effects of endocrine disrupting chemicals Annu. Rev. Environ. Resour. 42 105 160 10.1146/annurev-environ-102016-061111

[50] Mnif W. Hassine A.I.H. Bouaziz A. Bartegi A. Thomas O. Roig B 2011 Effect of endocrine disruptor pesticides: a review Int. J. Environ. Res. Public Health 8 2265 2303 10.3390/ijerph8062265 21776230 PMC3138025

[51] Raja G.L. Subhashree K.D. Kantayya K.E 2022 In utero exposure to endocrine disruptors and developmental neurotoxicity: Implications for behavioural and neurological disorders in adult life Environ. Res 203 111829 10.1016/j.envres.2021.111829 34358505

[52] Street M.E. Angelini S. Bernasconi S. Burgio E. Cassio A. Catellani C. et al. 2018 Current Knowledge on Endocrine Disrupting Chemicals (EDCs) from Animal Biology to Humans, from Pregnancy to Adulthood: Highlights from a National Italian Meeting Int. J. Mol. Sci. 19 1647 10.3390/ijms19061647 29865233 PMC6032228

[53] Streifer M. Gore A.C 2021 Epigenetics, estrogenic endocrine-disrupting chemicals (EDCs), and the brain Adv. Pharmacol 92 73 99 10.1016/bs.apha.2021.03.006 34452697

[54] Kahn L.G. Philippat C. Nakayama S.F. Slama R. Trasande L 2020 Endocrine-disrupting chemicals: implications for human health Lancet Diabetes Endocrinol 8 703 718 10.1016/S2213-8587(20)30129-7 32707118 PMC7437820

[55] Segovia-Mendoza M. Palacios-Arreola M.I. Pavón L. Becerril L.E. Nava-Castro K.E. Amador-Muñoz O. et al. 2022 Environmental pollution to blame for depressive disorder? Int. J. Environ. Res. Public Health 19 1737 10.3390/ijerph19031737 35162759 PMC8835056

[56] Palanza P. Gioiosa L. Vom Saal F.S. Parmigiani S 2008 Effects of developmental exposure to bisphenol A on brain and behavior in mice Environ. Res 108 150 157 10.1016/j.envres.2008.07.023 18949834

[57] Nagel S.C. Parmigiani S. Vom Saal F.S 2016 Perinatal exposure to endocrine disruptors: sex, timing and behavioral endpoints Curr. Opin. Behav. Sci. 7 69 75 10.1016/j.cobeha.2015.11.017 27019862 PMC4805122

[58] Patisaul HB 2024 Dirty Minds: How Endocrine Disrupting Chemicals (EDCs) and Other Pollutants Affect the Neuroendocrinology of Behavior and Emotions In Neuroendocrinology of Behavior and Emotions: Environmental and Social Factors Affecting Behavior ( Caldwell H.K. Albers H.E. eds pp 217 249 Springer International Publishing 10.1007/978-3-031-51112-7_9

[59] Kajta M. Wójtowicz AK 2013 Impact of endocrine-disrupting chemicals on neural development and the onset of neurological disorders Pharmacol. Rep. 65 1632 1639 10.1016/s1734-1140(13)71524-x 24553011

[60] Aljabali A.A.A. Alkaraki A.K. Gammoh O. Tambuwala M.M. Mishra V. Mishra Y. et al. 2024 Deciphering depression: epigenetic mechanisms and treatment strategies Biology (Basel) 13 638 10.3390/biology13080638 39194576 PMC11351889

[61] Chen W.-Q. Yuan L. Xue R. Li Y.F. Su R.B. Zhang Y.Z. et al. 2011 Repeated exposure to chlorpyrifos alters the performance of adolescent male rats in animal models of depression and anxiety Neurotoxicology 32 355 361 10.1016/j.neuro.2011.03.008 21453723

[62] Kundakovic M. Gudsnuk K. Franks B. Madrid J. Miller R.L. Perera F.P. et al. 2013 Sex-specific epigenetic disruption and behavioral changes following low-dose in utero bisphenol A exposure Proc. Natl. Acad. Sci. U.S.A. 110 9956 9961 10.1073/pnas.1214056110 23716699 PMC3683772

[63] Akanbi C.A. Rotimi D.E. Ojo A.B. Ojo O.A 2025 Endocrine-disrupting chemicals (EDCs) and epigenetic regulation in embryonic development: Mechanisms, impacts, and emerging trends Toxicol. Rep. 14 101885 10.1016/j.toxrep.2024.101885 40612660 PMC12223400

[64] Derghal A. Djelloul M. Trouslard J. Mounien L 2016 An emerging role of micro-RNA in the Effect of the endocrine disruptors Front. Neurosci. 10 318 10.3389/fnins.2016.00318 27445682 PMC4928026

[65] Kim K 2024 The role of endocrine disruption chemical-regulated aryl hydrocarbon receptor activity in the pathogenesis of pancreatic diseases and cancer Int. J. Mol. Sci. 25 3818 10.3390/ijms25073818 38612627 PMC11012155

[66] Zhang W. Xie H.Q. Li Y. Zhou M. Zhou Z. Wang R. et al. 2022 The aryl hydrocarbon receptor: A predominant mediator for the toxicity of emerging dioxin-like compounds J. Hazard. Mater. 426 128084 10.1016/j.jhazmat.2021.128084 34952507 PMC9039345

[67] Bock K.W 2020 Aryl hydrocarbon receptor (AHR)-mediated inflammation and resolution: Non-genomic and genomic signaling Biochem. Pharmacol 182 114220 10.1016/j.bcp.2020.114220 32941865

[68] Mackenzie Ross S.J. Brewin C.R. Curran H.V. Furlong C.E. Abraham-Smith K.M. Harrison V 2010 Neuropsychological and psychiatric functioning in sheep farmers exposed to low levels of organophosphate pesticides Neurotoxicol. Teratol. 32 452 459 10.1016/j.ntt.2010.03.004 20227490 PMC3042861

[69] Walker D.M. Gore A.C 2017 Epigenetic impacts of endocrine disruptors in the brain Front. Neuroendocrinol. 44 1 26 10.1016/j.yfrne.2016.09.002 27663243 PMC5429819

[70] Devi C.B. Reddy G.H. Prasanthi R.P.J. Chetty C.S. Reddy GR 2005 Developmental lead exposure alters mitochondrial monoamine oxidase and synaptosomal catecholamine levels in rat brain Int. J. Dev. Neurosci. 23 375 381 10.1016/j.ijdevneu.2004.11.003 15927761

[71] Roy B. Dunbar M. Agrawal J. Allen L. Dwivedi Y 2020 Amygdala-Based Altered miRNome and Epigenetic Contribution of miR-128-3p in Conferring Susceptibility to Depression-Like Behavior via Wnt Signaling Int. J. Neuropsychopharmacol. 23 165 177 10.1093/ijnp/pyz071 32173733 PMC7171932

[72] Perera F.P. Rauh V. Whyatt R.M. Tsai W.-Y. Tang D. Diaz D. et al. 2006 Effect of Prenatal exposure to airborne polycyclic aromatic hydrocarbonson neurodevelopment in the first 3 years of life among inner-city children Environ. Health Perspect. 114 1287 1292 10.1289/ehp.9084 16882541 PMC1551985

[73] Edwards S.C. Jedrychowski W. Butscher M. Camann D. Kieltyka A. Mroz E. et al. 2010 Prenatal exposure to airborne polycyclic aromatic hydrocarbons and children’s intelligence at 5 years of age in a prospective cohort study in Poland Environ. Health Perspect. 118 1326 1331 10.1289/ehp.0901070 20406721 PMC2944097

[74] Fitsanakis V.A. Au C. Erikson K.M. Aschner M 2006 The effects of manganese on glutamate, dopamine and gamma-aminobutyric acid regulation Neurochem. Int. 48 426 433 10.1016/j.neuint.2005.10.012 16513220

[75] Suzuki T. Mizuo K. Nakazawa H. Funae Y. Fushiki S. Fukushima S. et al. 2003 Prenatal and neonatal exposure to bisphenol-A enhances the central dopamine D1 receptor-mediated action in mice: enhancement of the methamphetamine-induced abuse state Neuroscience 117 639 644 10.1016/s0306-4522(02)00935-1 12617968

[76] Masuo Y. Ishido M 2011 Neurotoxicity of endocrine disruptors: possible involvement in brain development and neurodegeneration J. Toxicol. Environ. Health B. Crit. Rev. 14 346 369 10.1080/10937404.2011.578557 21790316

[77] Rauh V.A. Margolis A.E 2016 Research Review: Environmental exposures, neurodevelopment, and child mental health – new paradigms for the study of brain and behavioral effects Child Psychology Psychiatry 57 775 793 10.1111/jcpp.12537 PMC4914412 26987761

[78] Hou L. Zhang X. Wang D. Baccarelli A 2012 Environmental chemical exposures and human epigenetics Int. J. Epidemiol. 41 79 105 10.1093/ije/dyr154 22253299 PMC3304523

[79] Zhang L. Wang Z. Liu K. Li J. Li Y 2024 Investigation of the relationship between heavy metals in the blood and depression in people with different body mass indices using the NHANES database: A cross-sectional study J. Affect. Disord. 344 311 318 10.1016/j.jad.2023.10.023 37820959

[80] Orisakwe O.E 2014 The role of lead and cadmium in psychiatry N. Am. J. Med. Sci. 6 370 376 10.4103/1947-2714.139283 25210669 PMC4158644

[81] Pemberton R. Fuller Tyszkiewicz M.D 2016 Factors contributing to depressive mood states in everyday life: A systematic review J. Affect. Disord 200 103 110 10.1016/j.jad.2016.04.023 27131503

[82] Koller K. Brown T. Spurgeon A. Levy L 2004 Recent developments in low-level lead exposure and intellectual impairment in children Environ. Health Perspect. 112 987 994 10.1289/ehp.6941 15198918 PMC1247191

[83] Gump B.B. Dykas M.J. MacKenzie J.A. Dumas A.K. Hruska B. Ewart C.K et al. 2017 Background lead and mercury exposures: psychological and behavioral problems in children Environ. Res. 158 576 582 10.1016/j.envres.2017.06.033 28715786 PMC5562507

[84] Zeng X. Xu C. Xu X. Zhang Y. Huang Y. Huo X 2021 Elevated lead levels in relation to low serum neuropeptide Y and adverse behavioral effects in preschool children with e-waste exposure Chemosphere 269 10.1016/j.chemosphere.2020.129380 33383249

[85] Wegmann B. Tatemoto P. Miemczyk S. Ludvigsson J. Guerrero-Bosagna C 2023 Identification of potentially relevant metals for the etiology of autism by using a Bayesian multivariate approach for partially censored values Sci. Rep. 13 12622 10.1038/s41598-023-38780-9 37537167 PMC10400650

[86] Baccarelli A. Bollati V 2009 Epigenetics and environmental chemicals Curr. Opin. Pediatr. 21 243 251 10.1097/mop.0b013e32832925cc 19663042 PMC3035853

[87] Martinez-Zamudio R. Ha H.C 2011 Environmental epigenetics in metal exposure Epigenetics 6 820 827 10.4161/epi.6.7.16250 21610324 PMC3230540

[88] Senut M.C. Cingolani P. Sen A. Kruger A. Shaik A. Hirsch H. et al. 2012 Epigenetics of early-life lead exposure and effects on brain development Epigenomics 4 665 674 10.2217/epi.12.58 23244311 PMC3555228

[89] Fragou D. Fragou A. Kouidou S. Njau S. Kovatsi L 2011 Epigenetic mechanisms in metal toxicity Toxicol. Mech. Methods 21 343 352 10.3109/15376516.2011.557878 21495872

[90] Zeng Z. Huo X. Zhang Y. Hylkema M.N. Wu Y. Xu X 2019 Differential DNA methylation in newborns with maternal exposure to heavy metals from an e-waste recycling area Environ. Res. 171 536 545 10.1016/j.envres.2019.01.007 30763874

[91] Horvath S 2013 DNA methylation age of human tissues and cell types Genome Biol. 14 3156 R115 10.1186/gb-2013-14-10-r115 24138928 PMC4015143

[92] Dhingra R. Nwanaji-Enwerem J.C. Samet M. Ward-Caviness CK 2018 DNA Methylation Age-environmental influences, health impacts, and its role in environmental epidemiology Curr. Environ. Health Rep. 5 317 327 10.1007/s40572-018-0203-2 30047075 PMC6173330

[93] Van der Laan L. Cardenas A. Vermeulen R. Fadadu R.P. Hubbard A.E. Phillips R.V et al. 2022 Epigenetic aging biomarkers and occupational exposure to benzene, trichloroethylene and formaldehyde Environ. Int 158 106871 10.1016/j.envint.2021.106871 34560324 PMC9084243

[94] Ringrose L. Paro R 2001 Remembering silence Bioessays 23 566 570 10.1002/bies.1082 11462210

[95] Sales A.J. Biojone C. Terceti M.S. Guimarães F.S. Gomes M.V. Joca SR 2011 Antidepressant‐like effect induced by systemic and intra‐hippocampal administration of DNA methylation inhibitors Br. J. Pharmacol. 164 1711 1721 10.1111/j.1476-5381.2011.01489.x 21585346 PMC3230817

[96] Shi Y.G. Tsukada Y 2013 The discovery of histone demethylases Cold Spring Harb. Perspect. Biol. 5 a017947 10.1101/cshperspect.a017947 24003214 PMC3753710

[97] Xu J. Andreassi M 2011 Reversible histone methylation regulates brain gene expression and behavior Horm. Behav. 59 383 392 10.1016/j.yhbeh.2010.08.019 20816965 PMC3084016

[98] Chen Z.J. Tian L 2007 Roles of dynamic and reversible histone acetylation in plant development and polyploidy Biochimica et Biophysica Acta (BBA) - Gene Structure and Expression 1769 295 307 10.1016/j.bbaexp.2007.04.007 17556080 PMC1950723

[99] Dugué P.-A. Jung C.-H. Joo J.E. Wang X. Wong E.M. Makalic E. et al. 2020 Smoking and blood DNA methylation: an epigenome-wide association study and assessment of reversibility Epigenetics 15 358 368 10.1080/15592294.2019.1668739 31552803 PMC7153547

[100] Weaver I.C.G. Cervoni N. Champagne F.A. D’Alessio A.C. Sharma S. Seckl J.R. et al. 2004 Epigenetic programming by maternal behavior Nat. Neurosci. 7 847 854 10.1038/nn1276 15220929

[101] Albert P.R 2010 Epigenetics in mental illness: hope or hype? J. Psychiatry Neurosci. 35 366 368 10.1503/jpn.100148 20964959 PMC2964366

[102] Kripalani S. Pradhan B. Gilrain K.L 2022 The potential positive epigenetic effects of various mind-body therapies (MBTs): a narrative review J. Complement. Integr. Med. 19 827 832 10.1515/jcim-2021-0039 34463076

[103] Kaliman P. Cosín-Tomás M. Madrid A. Roque López S. Llanez-Anaya E. Papale L.A. et al. 2022 Epigenetic impact of a 1-week intensive multimodal group program for adolescents with multiple adverse childhood experiences Sci. Rep. 12 17177 10.1038/s41598-022-21246-9 36266402 PMC9585146

[104] Covington H.E. 3rd Maze I. LaPlant Q.C. Vialou V.F. Ohnishi Y.N. Berton O. et al. 2009 Antidepressant actions of histone deacetylase inhibitors J. Neurosci. 29 11451 11460 10.1523/JNEUROSCI.1758-09.2009 19759294 PMC2775805

[105] Toth M 2021 Epigenetic neuropharmacology: drugs affecting the epigenome in the brain Annu. Rev. Pharmacol. Toxicol. 61 181 201 10.1146/annurev-pharmtox-030220-022920 32997604 PMC9915026

[106] Falahi F. Van Kruchten M. Martinet N. Hospers G.A.P. Rots M.G 2014 Current and upcoming approaches to exploit the reversibility of epigenetic mutations in breast cancer Breast Cancer Res 16 412 10.1186/s13058-014-0412-z 25410383 PMC4303227

[107] Gladkova M.G. Leidmaa E. Anderzhanova E.A 2023 Epidrugs in the therapy of central nervous system disorders: a way to drive on? Cells 12 1464 10.3390/cells12111464 37296584 PMC10253154

[108] Czarny P. Białek K. Ziółkowska S. Strycharz J. Barszczewska G. Sliwinski T 2021 The importance of epigenetics in diagnostics and treatment of major depressive disorder J. Pers. Med. 11 167 10.3390/jpm11030167 33804455 PMC7999864

[109] Singh D. Khan M.A. Siddique H.R 2023 Role of epigenetic drugs in sensitizing cancers to anticancer therapies: emerging trends and clinical advancements Epigenomics 15 517 537 10.2217/epi-2023-0142 37313832

[110] Suraweera A. O’Byrne K.J. Richard DJ 2025 Epigenetic drugs in cancer therapy Cancer Metastasis Rev. 44 37 10.1007/s10555-025-10253-7 40011240 PMC11865116

[111] Cossío F.P. Esteller M. Berdasco M 2020 Towards a more precise therapy in cancer: Exploring epigenetic complexity Curr. Opin. Chem. Biol 57 41 49 10.1016/j.cbpa.2020.04.008 32480315

[112] Nie J. Liu L. Li X. Han W 2014 Decitabine, a new star in epigenetic therapy: the clinical application and biological mechanism in solid tumors Cancer Lett 354 12 20 10.1016/j.canlet.2014.08.010 25130173

[113] Robison S.K 2016 The political implications of epigenetics: emerging narratives and ideologies Polit Life Sci 35 30 53 10.1017/pls.2016.14 28134041

[114] Park C. Rosenblat J.D. Brietzke E. Pan Z. Lee Y. Cao B et al. 2019 Stress, epigenetics and depression: a systematic review Neurosci. Biobehav. Rev 102 139 152 10.1016/j.neubiorev.2019.04.010 31005627

